# Recurrent clonal radiotherapy-associated fibroepithelial polyp of the pharynx: do low grade radiogenic stromal tumours exist? Case report

**DOI:** 10.1007/s00428-025-04252-w

**Published:** 2025-09-11

**Authors:** Andrej Sirek, Ivana Bratic Hench, Jürgen Hench, Ilaria Alborelli, Markus W. Gross, Jens Jakscha, Patrick Schaller, Gideon Nagel, Daniel Baumhoer, Alexandar Tzankov

**Affiliations:** 1 Institute of Pathology, University Hospital BaselInstitute of Pathology, University Hospital Basel, Basel, Switzerland; 2 Department of Radiotherapy and Radiooncology, University Hospital Basel, Basel, Switzerland; 3 Ears Nose Throat (ENT) Centre, University Hospital Basel, Basel, Switzerland; 4 ENT Centre Dr. Schaller, Basel, Switzerland; 5 Centre for Internal Medicine and Geriatrics, Dr. Nagel, Basel, Switzerland

**Keywords:** Radiotherapy-associated polyp, Radiotherapy, PTEN, DNMT3A, TERT, Squamous cell carcinoma of the pharynx

## Abstract

There is a wide range of tissue changes that may arise after exposition to ionizing radiation. Most of these changes fall in the non-neoplastic category. Nevertheless, due to the damaging effect radiation has on the DNA, there is a risk of developing secondary tumours, usually high-grade sarcomas. Here, we present a case of an elderly man who developed recurrent fibroepithelial polyps of the pharyngeal mucosa. He had a history of ipsilateral squamous cell carcinoma of the tonsil 17 years before treated by complete surgical resection followed by adjuvant radiotherapy. Histologically, these polyps consisted of relatively well-defined, partially eroded mucosal projections with a fibro-myxoid and inflamed stroma lacking evident signs of malignancy. The 4th recurrence was submitted to DNA panel sequencing, which identified three pathogenic variants in the *PTEN*, *DNMT3A*, and *TERT* genes that were not present in the local normal tissue before radiotherapy. On further analysis, well-known and established radiation induced changes such as copy number (CN) alterations or a radiation signature were not detected. The clinical presentation and the mutations detected argue against a purely reactive, but rather a likely benign neoplastic process that is thought to have developed post-actinically, without clear evidence of malignancy (absence of atypia, no invasive growth, flat CN profile). This case report raises awareness of a possible association between radiotherapy and the subsequent development of a benign-appearing low-grade mucosal soft tissue neoplasm.

**Supplementary Information:**

The online version contains supplementary material available at 10.1007/s00428-025-04252-w.

## Introduction

Radiotherapy is used to treat a wide spectrum of medical conditions, most of all neoplasms following neoadjuvant and adjuvant treatment protocols. It is an ancillary method used to further increase the probability of tumour eradication. Consequently, many patients benefit from remission of their disease and prolonged survival [1].

The downside, as with various other therapies, are potential side effects, one of which is the induction of secondary neoplasms, mainly aggressive high-grade sarcomas and myeloid malignancies [1–5]. 

The following case report describes a patient who unexpectedly developed recurring fibroepithelial polyps within the field of radiation, requiring multiple surgical interventions.

## Material and methods

### Specimen collection

Patient characteristics are described in the “Interdisciplinary case presentation” section. Biopsy specimens were collected and processed as part of the standard diagnostic routine. Briefly, tissue resections were collected in 10% buffered formalin. After embedding into paraffin, the formalin-fixed and paraffin-embedded (FFPE) blocks were cut and stained with Haematoxylin and Eosin (H&E).

### DNA extraction from tissue biopsy

DNA extraction from biopsies was performed using the Maxwell RSC FFPE Plus DNA Kit on the Maxwell RSC48 device (Promega, USA) according to the manufacturer’s instructions. DNA was eluted in 50 µl of nuclease-free water and quantified with the Qubit dsDNA HS Assay Kit (Molecular Probes, Thermo Fisher Scientific, USA) on the Qubit Fluorometer 2.0 (Thermo Fisher Scientific, USA) according to the manufacturer’s instructions.

### Next generation sequencing (NGS) library preparation and sequencing

DNA originating from FFPE tissues was pre-treated with Uracil-DNA glycosylase (UDG, Thermo Fisher Scientific, USA). Targeted sequencing was performed with the help of commercial targeted NGS panels: Oncomine Comprehensive Assay Plus and Oncomine Childhood Cancer Assay (Thermo Fisher Scientific, USA). Library preparation was performed according to the manufacturer’s instructions (Thermo Fisher Scientific, USA). Libraries were purified with the Agencourt AmpureXP Beads (Beckman Coulter, USA) and quantified by help of the Ion Universal Quantitation Kit (Thermo Fisher Scientific, USA). FFPE libraries were diluted to 50 pM, loaded on Ion 550 chips by the Ion Chef instrument, and sequenced on S5 Prime instrument (Thermo Fisher Scientific, Torrent Suite v5.16.1). Raw data were automatically processed on the Ion Torrent Server v5.16.1 and aligned to a hg19 reference genome using the Torrent Alignment software. Variant calling was performed by help of Ion Reporter Oncomine Comprehensive Plus-w2.3-DNA-Single Sample, Oncomine Comprehensive Plus-w3.1-DNA-Single Sample, and Oncomine Childhood Cancer Research-w2.6-DNA-Single Sample workflows (Thermo Fisher, USA).

### Methylation analysis

DNA was bisulphite converted by EZ-DNA Lightning Methylation Kit (Zymo Research, USA) and analyzed by the Illumina Infinium Human Methylation Beadchip EPICv2 K microarray according to the manufacturer’s protocol (Illumina, USA). DNA was analyzed using various methylation classifiers [6–8]. Data comparison was performed by UMAP algorithm based on TCGA and other published reference collectives [7].

### HPV genotyping

HPV genotyping for the following genotypes—high risk (16, 18, 26, 31, 33, 35, 39, 45, 51–53, 56, 58, 59, 66, 68, 69, 73, 82) and low risk (6, 11, 40, 42–44, 54, 61, 70)—was performed using the CE-IVD certified Allplex™ HPV28 Detection rtPCR Assay (Seegene). The Ct value is categorized on a three-level scale: + (Ct value < 35–43)/+ + (Ct value 25–34)/+ + + (Ct value < 25) = low/medium/high.

## Interdisciplinary case presentation

### Clinical aspects

**Fig. 1:**
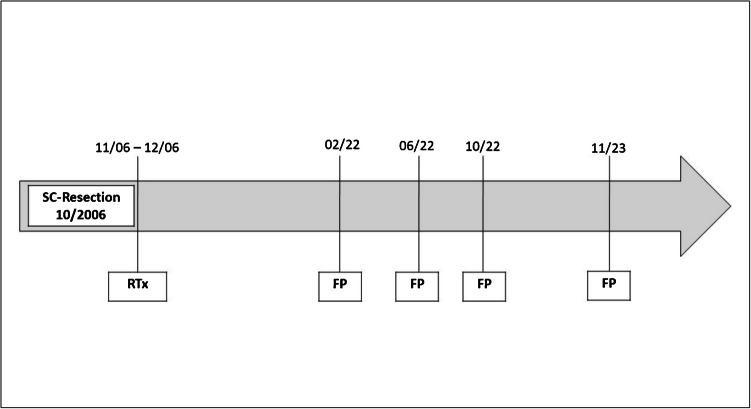
Timeline of initial radiotherapy treatment and the following development of fibroepithelial lesions. SC, squamous (cell) carcinoma; RTx, radiotherapy; FP, fibroepithelial polyp

An 83-year-old male presented with recurrent polypoid fibroepithelial lesions of the left lateral pharyngeal mucosa for the last 2 years (Fig. 1). The past medical history was significant for HPV-related squamous cell carcinoma (SCC) of the left palatine tonsil. Additional chronic ailments included gastroesophageal reflux disease associated with hiatal hernia, lactose intolerance, arterial hypertension, coronary artery disease, and hypothyroidism.

Seventeen years before, the patient presented with SCC of unknown primary as a histologically proven left cervical lymph node metastasis. Based on the PET scan and pan-endoscopy, there was a strong suspicion of an ipsilateral tonsillar carcinoma. After tonsillar biopsy-based confirmation of an HPV-associated SCC, the treatment of choice was tonsillectomy and neck dissection (level Ib-IV). Histologic evaluation of the tonsillar specimen revealed a 21-mm tumoral mass and 2 locoregional lymph node metastases (final TNM-classification: pT2, pN2b, G3, R0). HPV testing has not been done at the time of initial diagnosis but turned out to be positive for high-risk HPV on a later-on workup (data not shown in detail). Due to the lymph node metastasis, adjuvant radiotherapy was indicated.

Radiotherapy was performed over a period of 6 weeks with a percutaneous dose of 64 Gray (Gy), a lymph node drainage area dose of 56 Gy, and a supraclavicular lymph node area dose of 50.4 Gy. The treatment was successful, with an ongoing complete remission for 17 years.

Fifteen years after primary treatment, the patient complained of a relatively slow growing painless prominence in the left lateral wall of the pharynx that eventually led to discomfort. Clinically and radiologically, a recurrence of the previously diagnosed SCC was suspected.

### Endoscopy and macroscopic appearance

**Fig. 2:**
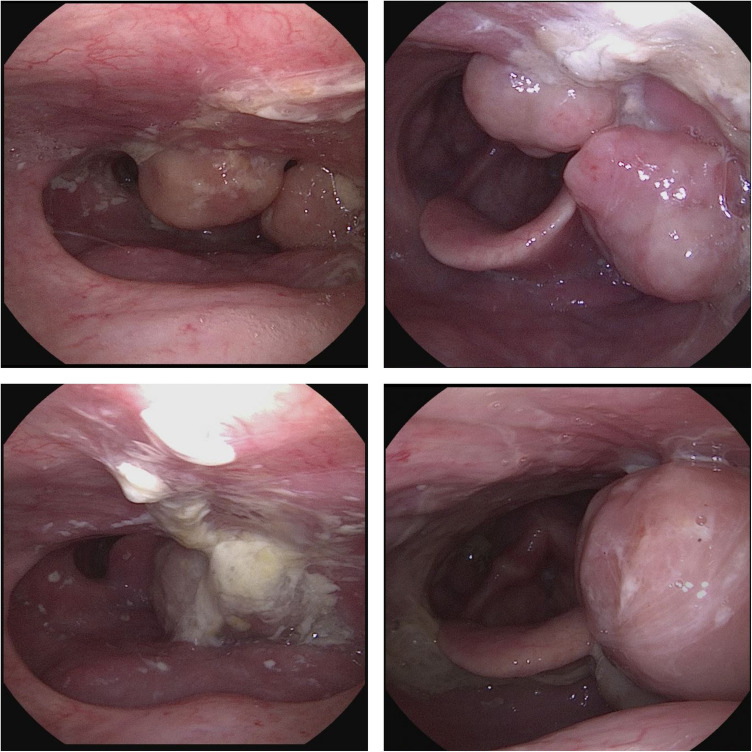
Endoscopic nasopharyngeal view of the fibroepithelial lesions. Left upper corner: initial presentation; right upper corner: 1st recurrence; left lower corner: 2nd recurrence; right lower corner: 3rd recurrence

Inspection of the 1st polypoid lesions revealed three 18–22 mm measuring and well-circumscribed masses with a relatively smooth pink-brown mucosal surface and narrow tissue pedicles blending into the left lateral oropharyngeal wall (Fig. 2). When attempting to biopsy the lesions, extirpation in toto occurred automatically, leaving behind a mucosal shell. After haemostasis at the site of biopsy and with an unremarkable pharyngoscopy, the procedure was completed without complications. The frozen section did not show evidence of malignancy.

On gross examination, the tissue was elastic-hard, the cut surface solid, white to beige.

The 1st and 2nd recurrences showed 2 lesions each, the last recurrence 1, ranging from 18 to 25 mm in diameter, and all of them shared similar macroscopic aspects and occurred at the same site.

### Histology

**Fig. 3:**
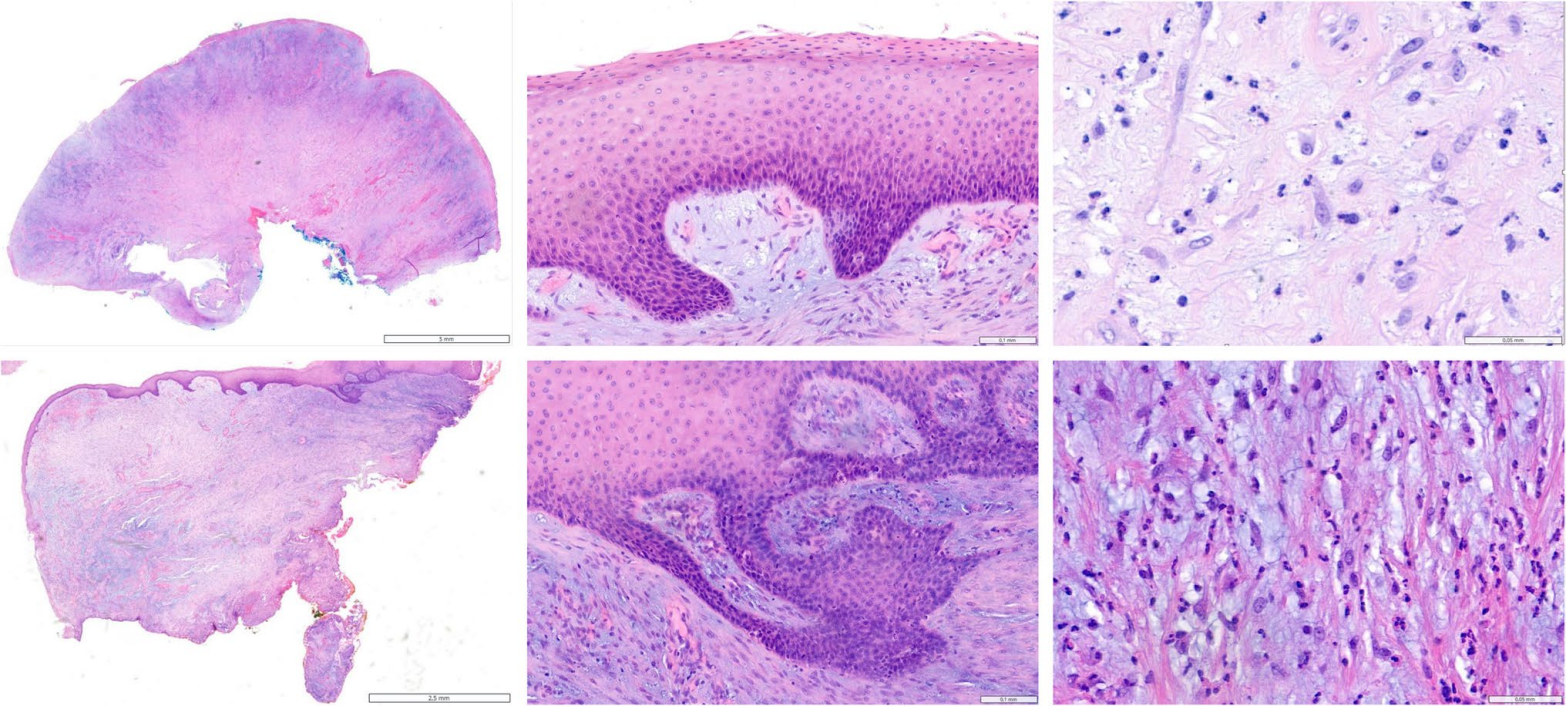
Histology (H&E) of the polyp: low power view of polypoid lesions with an abundance of subepithelial and myxoid appearing stroma (left). Closer view of non-keratinized, stratified squamous epithelium with minimal reactive atypia (middle), and subepithelial dense collagen stroma with myxoid changes, inflammatory cells, and myofibroblasts (right)

On H&E staining, the morphology was mainly identical in each of the manifestations—polypoid, nodular-shaped mucosal tissue covered with partially denuded and eroded squamous epithelium, with some areas of pseudoepitheliomatous hyperplasia, focal signs of reactive atypia, and lack of both dysplasia and invasive carcinoma (Fig. 3, SupplementH&E1-SupplementH&E16). The stroma below the epithelium was abundant and showed moderate chronic and active inflammation and myxoid aspects suggestive of degenerative change. No obvious signs of malignancy (e.g. nuclear pleomorphism, hyperchromasia, increased mitoses) were observed. The features were consistent with an unusual though reactive fibroepithelial polyp. To rule out mycosis, PAS stains were repeatedly performed, without any evidence of fungi or other stainable agents.

### Immunohistochemistry

Additional immunophenotyping showed no expression of ALK and ROS1. S100, CK22, and CD34 stained preexisting structures only. With smooth muscle markers, myofibroblastic cells were labelled within the inflammatory stroma. The proliferation index (Ki-67) was approximately 15%.

### Molecular analysis

DNA-based targeted NgS from the 4th recurrence using the Oncomine Comprehensive Assay Plus covering 498 genes yielded the following result with three pathogenic variants: *PTEN*: p.Q171* in exon 6; *DNMT3A*: p.P262Lfs*54 in exon 7; and *TERT*: c.−146C > T in the promoter (also described as C250T). The remaining variants with unclear oncogenic potential consisted of the following: *C8B*: p.S55N in exon 2; *NOTCH1*: p.N718S in exon 13; *ARID1B*: p.T2124M in exon 21; *KDM6A*: p.K799R in exon 17; *OR5L1*: p.P167L in exon 1; *SDHA*: p.E291Q in exon 7; and *TET2*: p.P101R in exon 3 (Table 1). After removing germline mutations, the detected somatic single nucleotide variants were used to create a normalized single base substitution (SBS) matrix or “mutational signature” using a plugin within the IonReporter (5.18) software. The normalized mutational signature of the patient sample was then compared with 54 signatures from the COSMIC Mutational Signatures v3.1, and the cosine similarity was calculated [9]. COSMIC signatures with cosine similarity score ≥ 0.7 to the sample signature were then analyzed with the deconstructSigs algorithm to determine which COSMIC Mutational Signatures were the best match. No unequivocal radiation signature was detected.

**Table 1:** DNA-based NGS gene variants found within the fibroepithelial polyp. Top table with relevant genetic variants with known pathogenic role, lower table with genetic variants of currently unknown significance

Gene	Locus	Transcript	Encoding	Amino acid replacement	Allele frequency
Relevant genetic variants					
*DNMT3A*	*ch2:25470975*	*NM_022552.5*	*c.785delC*	p.(P262Lfs*54)	4.18%
*PTEN*	*Chr10:89711893*	*NM_000314.8*	*c.511C* > *T*	p.(Q171*)	21.39%
*TERT*	*Chr5.1295250*	*NM_198253.2*	*c.146C* > *T*	*p.(?)*	8.05%
Genetic variants of unknown significance					
*C8B*	*chr1:57425778*	*NM_000066.4*	*c.164G* > *A*	*p.(S55N)*	50.43%
*TET2*	*chr4:106155401*	*NM_001127208.3*	*c.302C* > *G*	*p.(P101R)*	9.26%
*SDHA*	*chr5:231091*	*NM_004168.4*	*c.871G* > *C*	*p.(E291Q)*	9.85%
*ARID1B*	*chr6:157528397*	*NM_001371656.1*	*c.6371C* > *T*	*p.(T2124M)*	42.72%
*NOTCH1*	*chr9:139409016*	*NM_017617.5*	*c.2153A* > *G*	*p.(N718S)*	10.10%
*OR5L1*	*chr11:55579442*	*NM_001004738.2*	*c.500C* > *T*	*p.(P167L)*	44.95%
*KDM6A*	*chrX:44929296*	*NM_021140.3*	*c.2396A* > *G*	*p.(K799R)*	4.32%

To assign the mutations to the stromal and/or epithelial components, we performed enrichment experiments by scratching off the epithelial-devoid pure stromal component (Supplementary Fig. 1) with a lancet, and by laser microdissection of the epithelial component with some minimal amounts of intervening stroma in the papillary tips of the lamina propria (Supplementary Fig. 2) to finally extract DNA for sequencing of either component.

Analysis of the stromal component confirmed the presence of the same variants identified in the initial examination, specifically *PTEN* with a variant allele frequency (VAF) of 27% (initially 21%) and *TERT* with a VAF of 19% (initially 8%), as well as the *TET2* variant (12 and 9%, respectively).

Analysis of the epithelial component detected the known *PTEN* variant with a VAF of 28%. However, due to low DNA quality, the other variants could not be reliably determined.

**Fig. 4:**
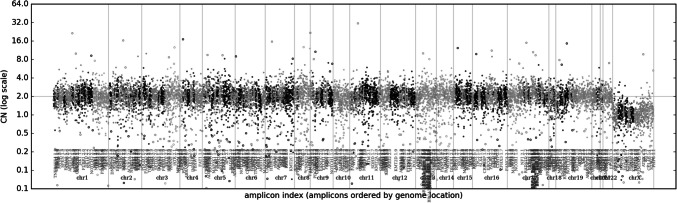
Copy number profile found within the polyp: flat profile

No copy number alterations of the sequenced loci were detected (Fig. 4). The tumour mutation burden was low: 3.8 variants per megabase. The microsatellite instability score indicated a stable state: 3.9. The genomic instability metric was low (GIM-Score): 1.

### Additional testing

RtPCR–HPV failed to detect virus DNA in the sample from the 4th recurrence.

An RNA-based panel NgS of the 4th recurrence using the Archer FusionPlex Pan Solid Tumour v2 Panel covering 137 genes was performed to investigate possible gene fusions. There was no evidence of any gene fusions.

A methylome analysis in the polyp sample of the 4th recurrence using various methylation classifiers [6–8] did not yield any additional diagnostic findings, and the hereby copy number analysis confirmed a flat copy number profile as previously suggested by the targeted sequencing (Fig. 4).

### Comparative analysis

To examine whether the above discovered mutations were private to the fibroepithelial polyps or already present in the SCC or in the unaffected non-tumorous patient’s tissues prior to radiotherapy, a DNA-based targeted NgS on the archived tissue blocks was performed, both with and without SCC, that were collected 17 years ago. The pathological variants detected in the fibroepithelial polyp were neither present in the SCC nor in the healthy, unexposed tissue. The *ARID1B:* p.T2124M variant was present in all, SCC and normal tissue, as it was in the fibroepithelial polyp, likely representing a germline variant.

### Outcome and follow-up

Before molecular testing of the sample of 3rd recurrence, descriptive diagnosis (squamous epithelium with focal atypia, subepithelial inflammation, and myxoid sclerosis), compatible with an unusual fibroepithelial polyp, was provided. The results of DNA sequencing of the 4th recurrence, showing several mutations in known tumour suppressor genes, spoke against an exclusively reactive process and favoured a neoplastic one. In the context of the morphology, no malignant tumour diagnosis could be established. Thus, the descriptive diagnosis was modified to benign-appearing low-grade mucosal soft tissue neoplasm, not further classifiable, advising the patient to be under close clinical observation.

At the time of writing this report, 1 year after the last polypoid lesion was excised, the patient is free of disease.

The consequence of the multiple surgical interventions, however, led to a significantly decreased quality of life—velopharyngeal insufficiency, lower sensitivity of the anterior tongue, and reduced mouth opening due to pain translated to hypernasal speech (*rhinophonia aperta*) and difficulty of speaking; severe dysphagia led to repeated choking while eating and drinking and weight loss. Through logopaedic therapy, the patient was able to improve his ability to eat and drink.

## Discussion

Our case report presents an elderly male patient developing recurrent polypoid fibroepithelial lesions in a previously irradiated tissue/field. It is an investigation of the pathogenesis behind what was initially considered a reactive lesion.

Despite the suggestive clinical behaviour of the lesions (recurrent local tumour growth), histomorphologically, no malignant tumour could be diagnosed. Here, we demonstrate that the molecular analyses can lead to a better understanding of the nature of such lesions and allow a more precise designation, while refined classification is still impossible, as no WHO-recognized category—except for fibroepithelial polyp in Cowden syndrome (see later)—exists.

Through DNA-based targeted NGS, we were able to detect several mutations in known tumour suppressor genes, including *PTEN*. These findings argue against a purely reactive lesion and favour a neoplastic process. After comparing the DNA from the fibroepithelial polyp with that of the healthy appearing tissue prior to radiation therapy, it was apparent that the *PTEN* mutation came to be a de novo event.

With respect to the epithelial and stromal sub-compounds of the polyps, we demonstrated the presence of the *PTEN* mutation in the stromal compartment, accompanied by an enrichment of the *TERT* variant after macro-/microdissection. The analysis of the microdissected epithelial component, however, was limited by poor DNA quantity and quality, further complicated by unavoidable contamination from stromal cells due to the presence of papillary projections of the underlying stroma within the dissection material, and yielded detection of the *PTEN* mutation only. Thus, while the *PTEN* and the *TERT* mutations could unequivocally be assigned to the stromal compound, their presence in the epithelium could not be confirmed or excluded beyond doubt.

The enrichment of the *TERT* variant in the stromal component after macrodissection is particularly notable given TERT’s established role in tumour biology. Increased TERT expression, being the net effect of *TERT* promoter mutations, leads to elevated telomerase activity, which enables cells to escape replicative senescence and promotes sustained cellular proliferation—key mechanisms implicated in tumour progression and maintenance [10]. To this end, all our findings suggest that the stromal compartment had most significantly contributed to the oncogenic processes.

Based on the clinical history, the histopathological findings, and the molecular data, the lesion is mostly reminiscent of a local Cowden syndrome-equivalent due to a mutation of the *PTEN* gene in the context of radiotherapy. Indeed, the clinical manifestation of *PTEN* hamartoma tumour syndrome in the oropharyngeal region is that of multiple mucosal papillomatoses, nodular gingival hyperplasia, and fibroepithelial polyps [11–13]. *PTEN* mutations typically do not develop directly because of radiation therapy; however, radiation exposure can increase the likelihood of genetic mutations in general, including *PTEN* mutations, over time [14].

The other mutations found in our case (*DNMT3A*, *C8B*, *TET2*, *SDHA*, *ARID1B*, *NOTCH1*, *OR5L1*, and *KDM6A*) do not have a known association with the formation of fibroepithelial polyps. *ARID1B* mutations have been implicated in certain types of cancers, such as ovarian and breast, and—more recently—endometrial carcinomas and renal tumours. While *ARID1B* mutations may not directly cause polyps, mutations in chromatin remodelling genes (such as *ARID1B*) can contribute to genomic instability, which may play a role in the development of cancerous or precancerous lesions [15].

Radiation-induced neoplasms are generally associated with certain characteristic genetic alterations (like chromosomal translocations or *TP53* mutations) and exhibit characteristic mutational signatures. Such changes were not detected in the lesion presented here. However, there are cases in which respective post-radiotherapy neoplasms do not display classic signatures. This can occur due to a variety of factors, including delayed onset, genomic instability, and additional environmental exposures that may alter the mutational profile of the lesion [16]. Hence, it is possible to have radiation-induced neoplasms that do not exhibit clear and typical genetic alterations [17].

Clearly, irradiation causes tissue changes in the affected area, leading to inflammation, fibrosis, vascular changes, and occasionally development of malignant tumours [18, 19]. Concerning the head and neck region, the risk of developing secondary malignancies is well established [20–27]. Conversely, tumour-like or benign lesions associated with radiotherapy in general have been described in the literature, including fibroepithelial polyps of the urinary bladder, the vulva, and the penis [28–30]. Polypoid lesions following radiation therapy of oral cavity SCC have been described in the floor of the mouth, gingiva, and tongue. The majority have been interpreted as reactive pseudotumours showing fibroblastic appearing spindle cells that can mimic secondary sarcoma but showing a favourable prognosis in the majority of cases [31]. Likewise, there are reports of non-malignant polyps and masses of the nasopharynx and sphenoid sinus that developed after radiotherapy for nasopharyngeal carcinoma—histologically showing granulation tissue with variable amounts of fibrin and inflammatory cells [32, 33].

It is important to consider that the lesion described in our report shows partial morphological overlap with a relatively commonly encountered mucosal change of the oropharyngeal region—a reactive hyperplastic outgrowth classified as traumatic/irritation fibroma, pyogenic granuloma, and fibrous hyperplasia [34]. Fibroepithelial polyp, a term interchangeably described as irritation fibroma without association to radiotherapy, is frequently encountered as a mucosal nodule intraorally, in the tonsil and in the nasal vestibule [13, 35–43]. The development of such lesions has been correlated with age, and an increased fragility of the mucosa with reduced immunological reactivity and impaired DNA repair/regeneration might contribute pathogenetically. The other factors that play a role include trauma, medications, and poor oral hygiene [44]. These lesions are usually solitary, and upon surgical intervention, their recurrence is rare [45].

Lastly, in the histological differential diagnosis of morphologically bland appearing spindled lesions with various amounts of inflammation and myxoid stroma, one should consider epithelial (e.g. spindle cell carcinoma), melanocytic (e.g. desmoplastic melanoma), and soft tissue neoplasms such as inflammatory myofibroblastic tumour, desmoid fibromatosis, or solitary fibrous tumour [46–48]. Ordering a limited panel of immunohistochemical stains for cytokeratins, S100, CD34, ALK, ROS1, STAT6, beta-catenin, and p16 will help to rule out these differential diagnoses.

## Conclusion

Tissue irradiation can cause genomic instability, potentially triggering the development of abnormal growths, including fibroepithelial polyps, though this is not commonly noted in clinical practice [49, 50]. In the presented case, it seems highly presumable that the radiation therapy caused these outgrowths and the DNA alterations identified. All the recurring outgrowths arising in the irradiation field, the normal Surrounding tissue not exhibiting the mutations, and the 15-year latency further Support this hypothesis. When encountering Such lesions, particularly after the 1st recurrence, the clinical outcome of our patient suggests that it might be advisable to undertake surgical excision early in the disease course. Furthermore, broader excision margins, increasing the probability of complete removal and thus preventing further recurrences with additional surgeries and associated complications, seem to be reasonable. A diagnostic molecular testing in the form of NGS on analogous lesions should be considered early as it might help to differentiate reactive, and probably not prone to recur, from neoplastic lesions that will highly likely recur, if incompletely resected, and improve further treatment planning. Even though there is no extensive evidence linking radiotherapy with the development of clonal fibroepithelial polyps, radiation-induced (or even spontaneous) mutations of genes involved in hamartomatous disorders such as *PTEN *may evolve in and imitate benign proliferations of known germline syndromes such as Cowden; indeed, analogous disorders may be due to germline or acquired mutations of the same genes, e.g. inborn and acquired autoimmune lymphoproliferative syndromes (ALPS) [51–60]. Further research may help to clarify whether fibroepithelial polyps can be considered radiation-induced benign neoplastic lesions. However, based on current knowledge, this remains an anecdotally observed outcome in the broader context of radiation-induced changes.

## Supplementary Information

Below is the link to the electronic supplementary material.

ESM 1 (JPG 2.56 MB)

ESM 2 (JPG 3.03 MB)

ESM 3 (JPG 2.91 MB)

ESM 4 (JPG 2.21 MB)

ESM 5 (JPG 2.85 MB)

ESM 6 (JPG 3.93 MB)

ESM 7 (JPG 4.00 MB)

ESM 8 (JPG 4.46 MB)

ESM 9 (JPG 3.49 MB)

ESM 10 (JPG 3.04 MB)

ESM 11 (JPG 4.25 MB)

ESM 12 (JPG 5.00 MB)

ESM 13 (JPG 4.55 MB)

ESM 14 (JPG 5.41 MB)

ESM 15 (JPG 4.09 MB)

ESM 16 (JPG 3.48 MB)

ESM 17 (JPG 7.23 MB)

ESM 18 (JPG 6.30 MB)

## Data Availability

Additional (particularly) sequencing data can be obtained from the authors upon personal request.
