# Supplementary figures and images for: Recurrent clonal radiotherapy-associated fibroepithelial polyp of the pharynx: do low grade radiogenic stromal tumours exist? Case report
Source: Virchows Arch. 2025 Sep 11;487(6):1407–15. doi: 10.1007/s00428-025-04252-w (PMC12748111; doi:10.1007/s00428-025-04252-w)

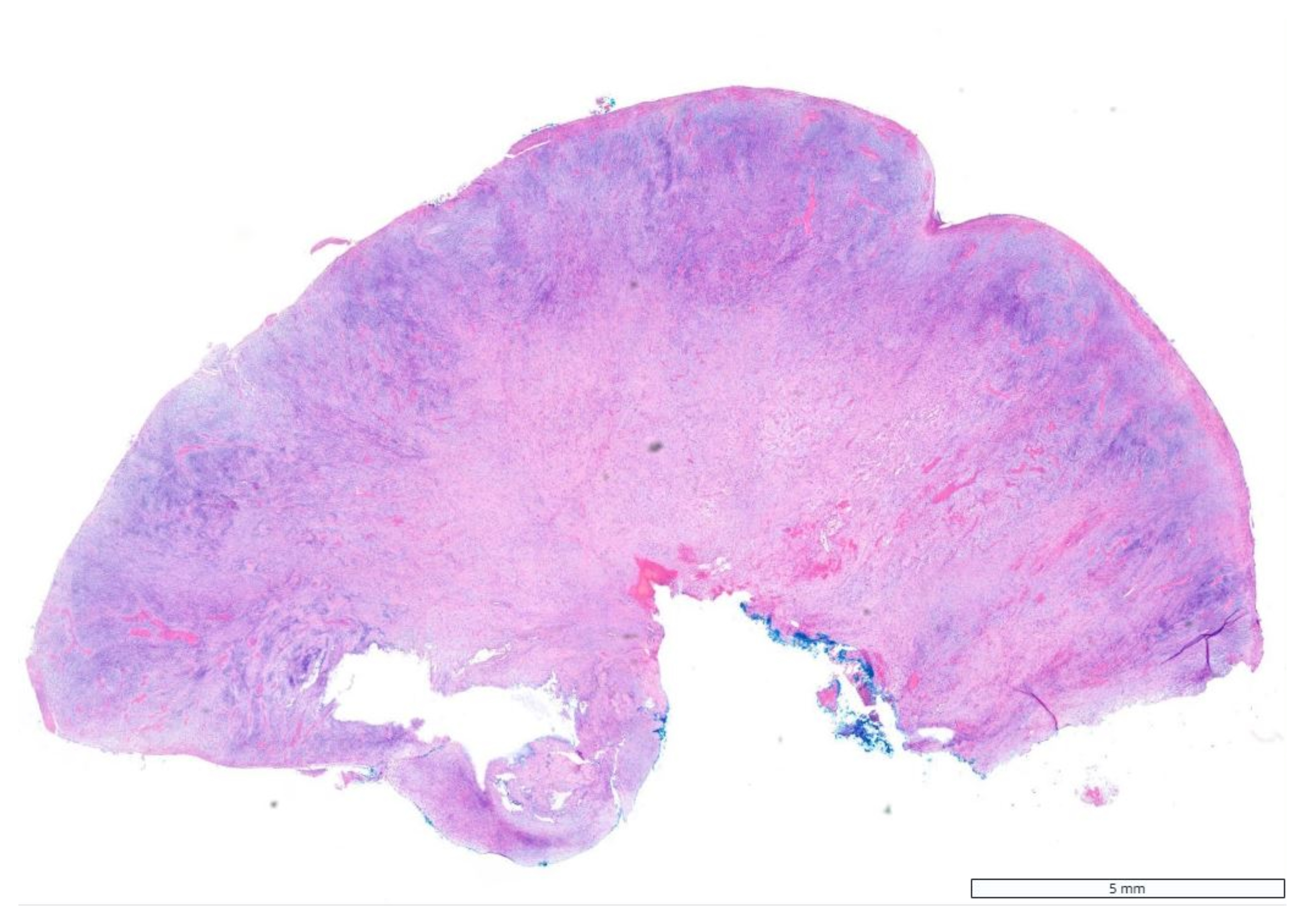

Supplement: Supplementary file 1 — (JPG 2.56 MB) [file 428_2025_4252_MOESM1_ESM.jpg]

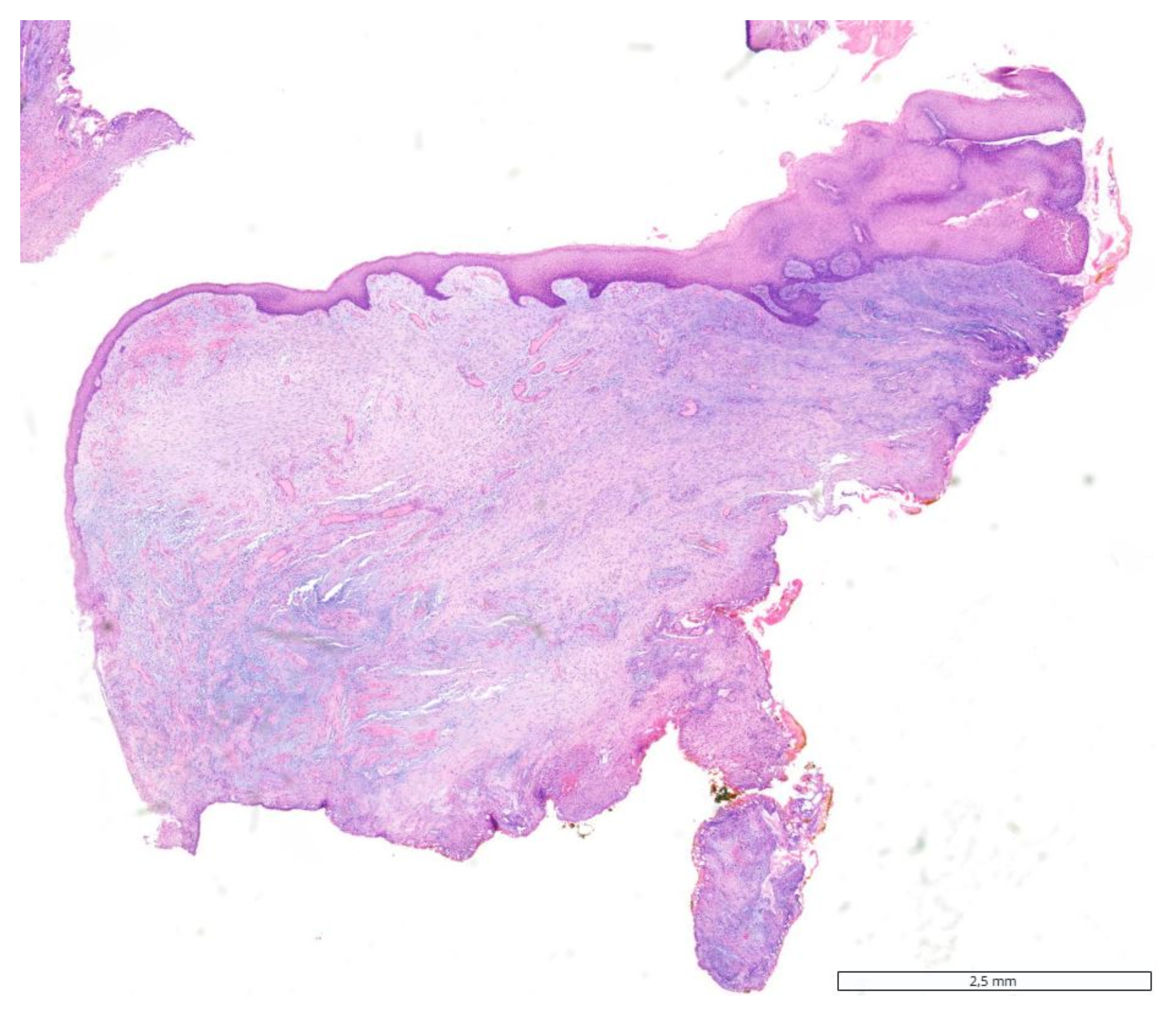

Supplement: Supplementary file 2 — (JPG 3.03 MB) [file 428_2025_4252_MOESM2_ESM.jpg]

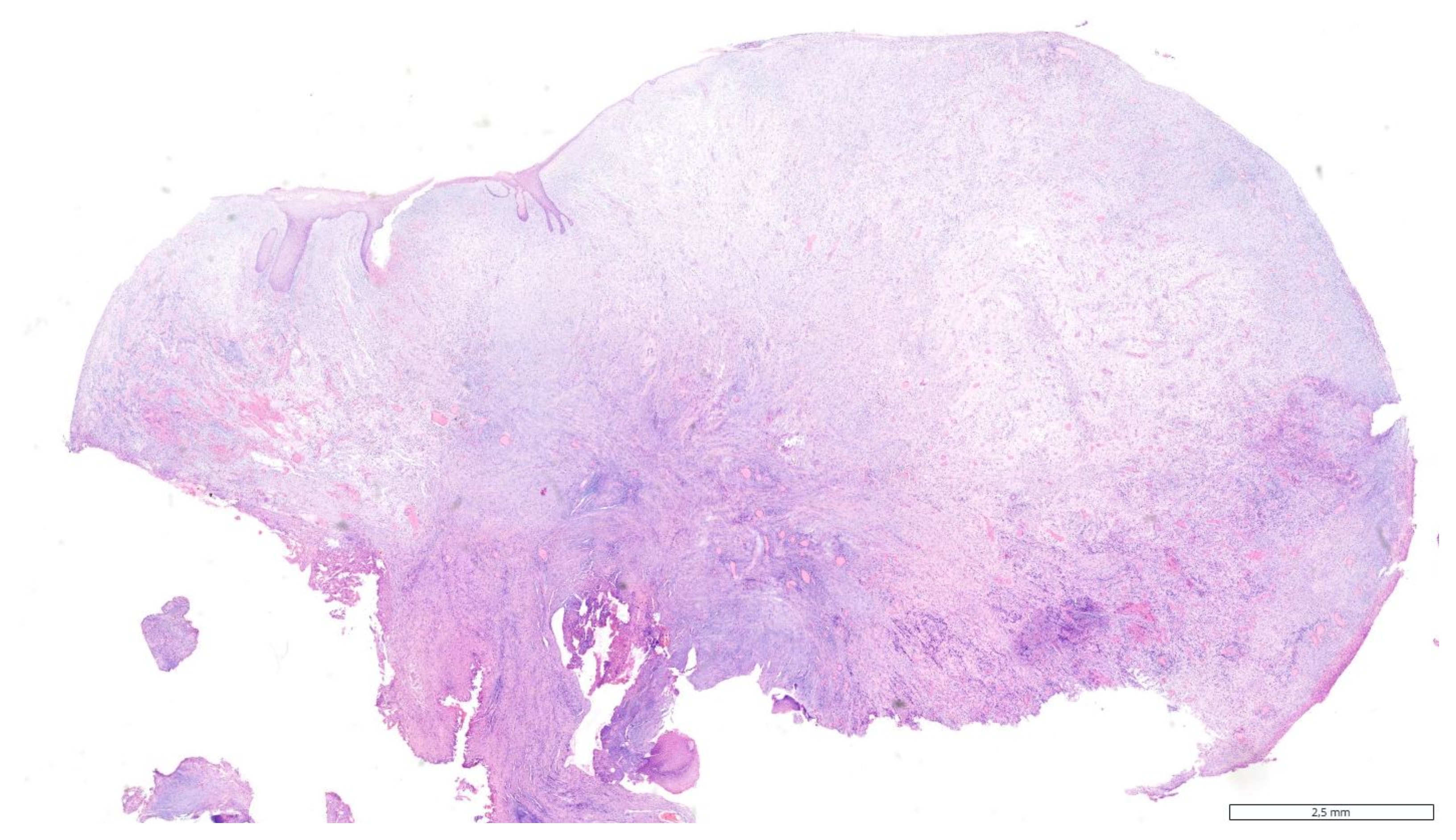

Supplement: Supplementary file 3 — (JPG 2.91 MB) [file 428_2025_4252_MOESM3_ESM.jpg]

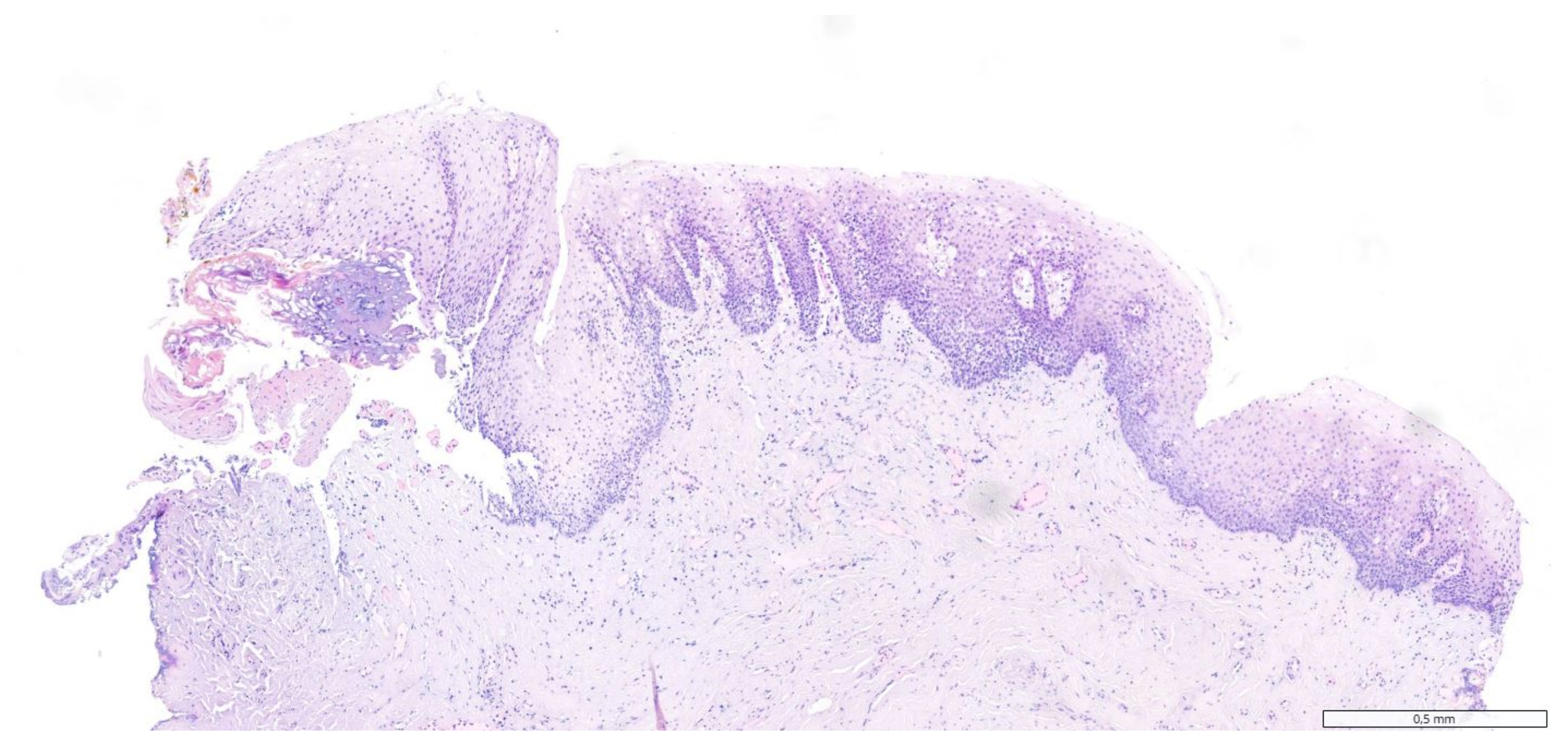

Supplement: Supplementary file 4 — (JPG 2.21 MB) [file 428_2025_4252_MOESM4_ESM.jpg]

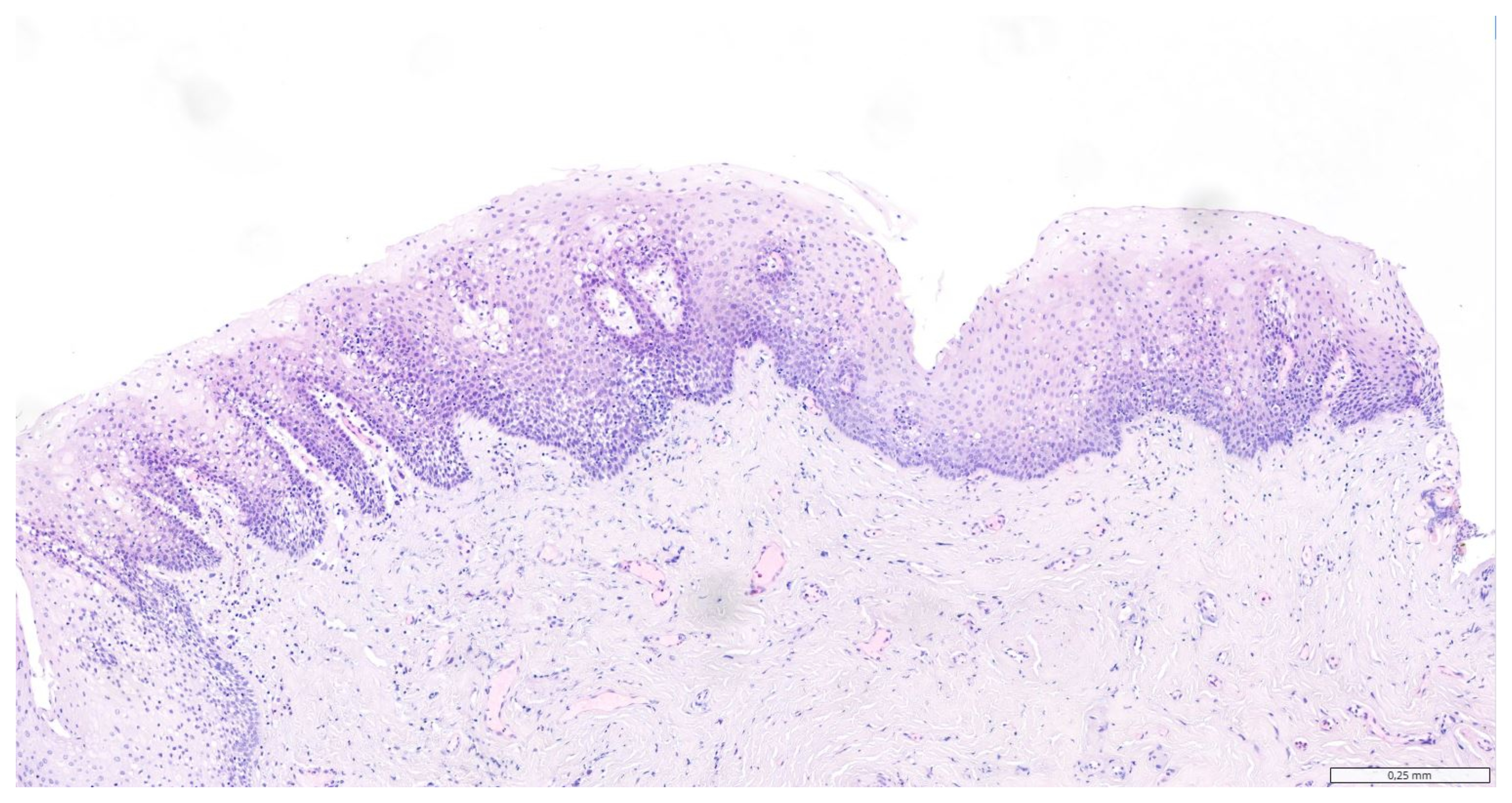

Supplement: Supplementary file 5 — (JPG 2.85 MB) [file 428_2025_4252_MOESM5_ESM.jpg]

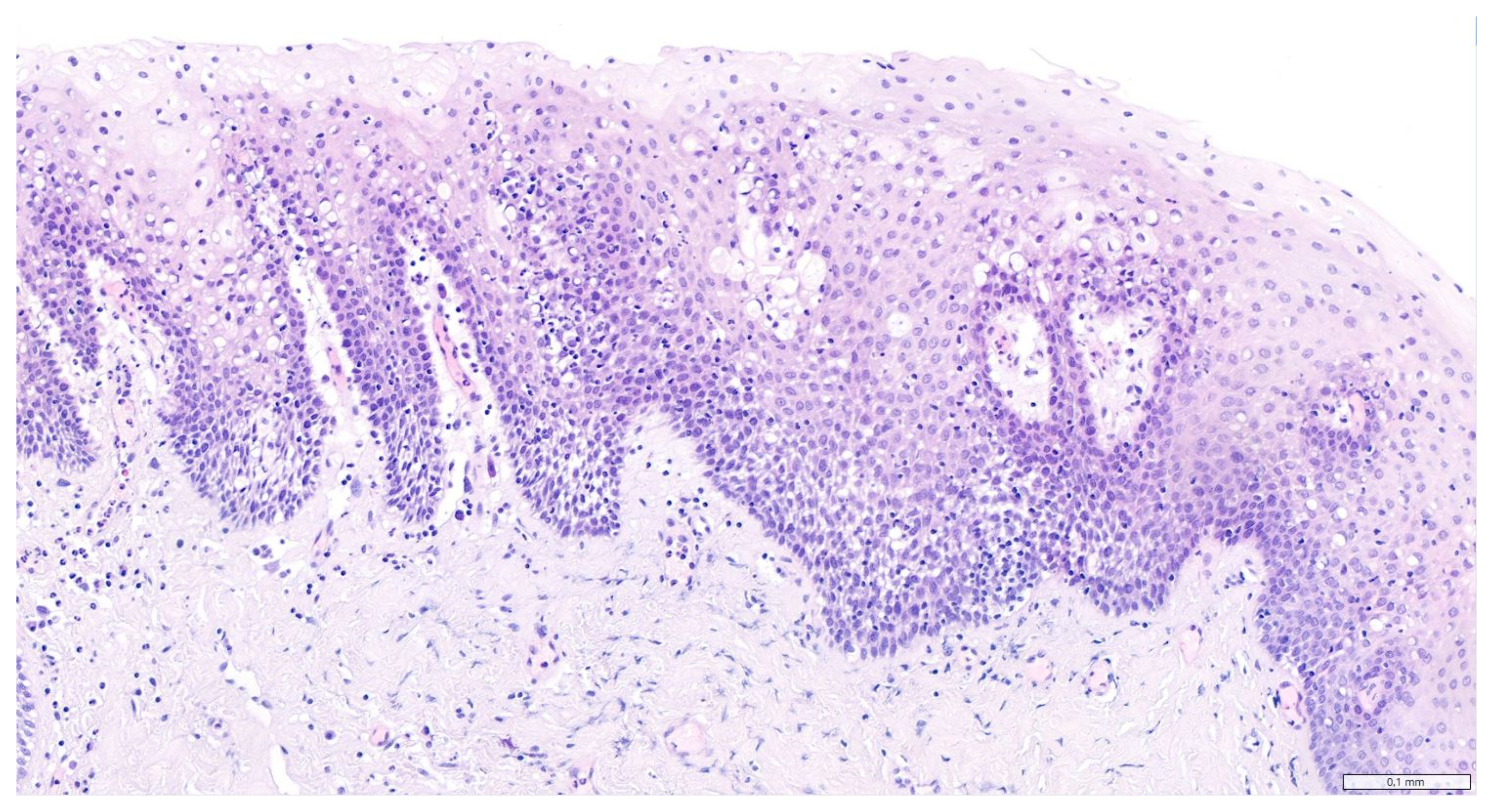

Supplement: Supplementary file 6 — (JPG 3.93 MB) [file 428_2025_4252_MOESM6_ESM.jpg]

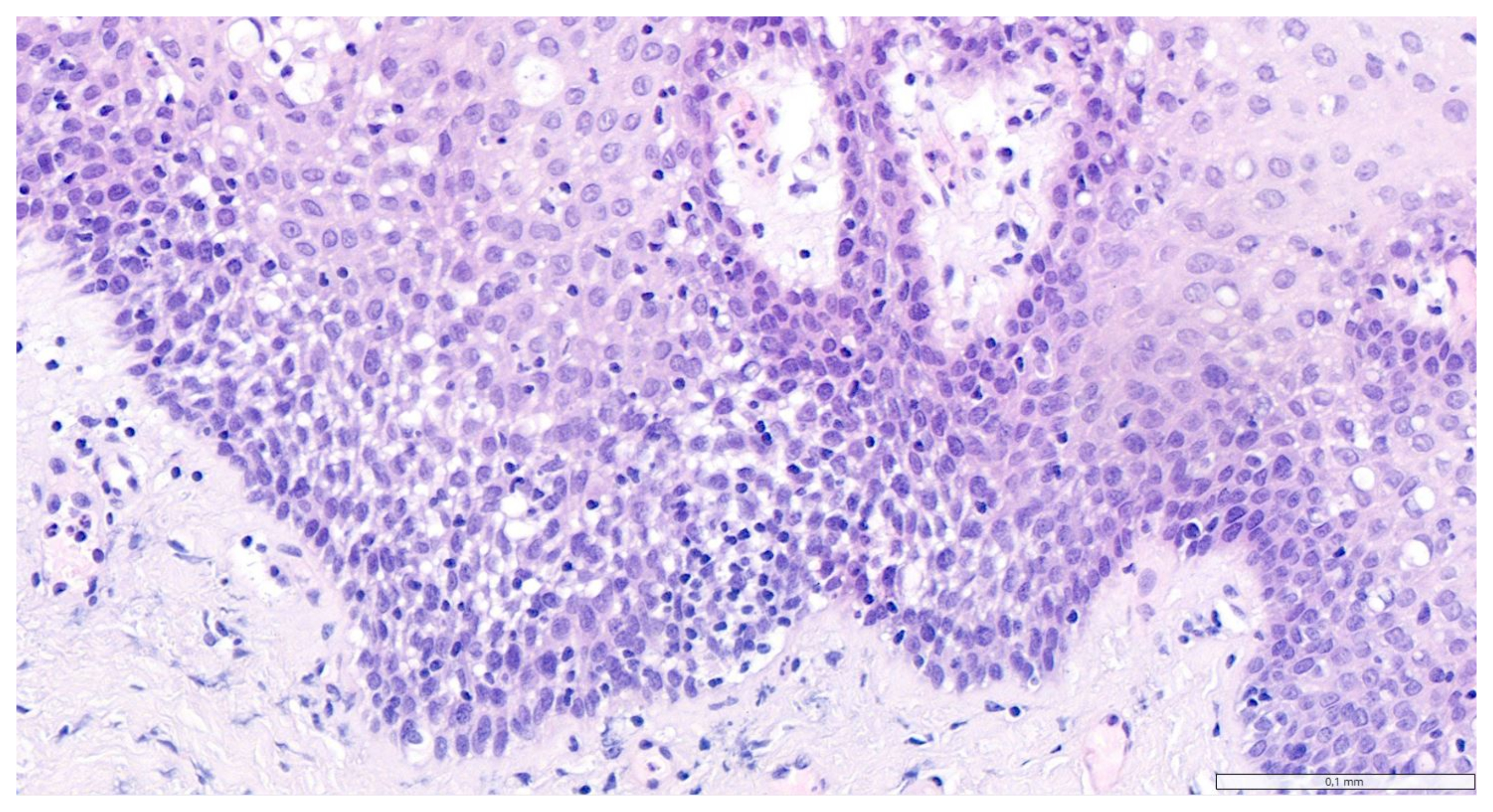

Supplement: Supplementary file 7 — (JPG 4.00 MB) [file 428_2025_4252_MOESM7_ESM.jpg]

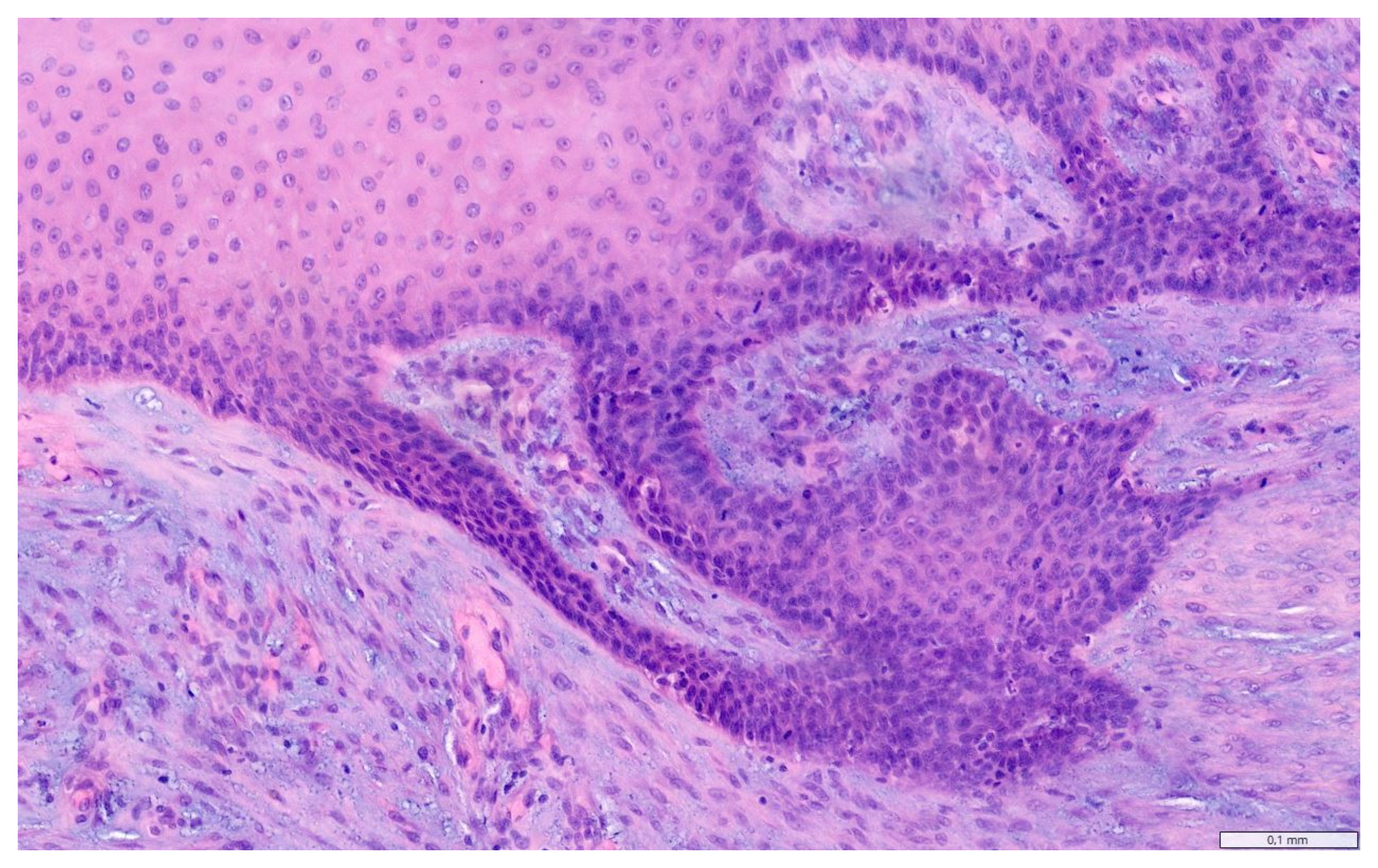

Supplement: Supplementary file 8 — (JPG 4.46 MB) [file 428_2025_4252_MOESM8_ESM.jpg]

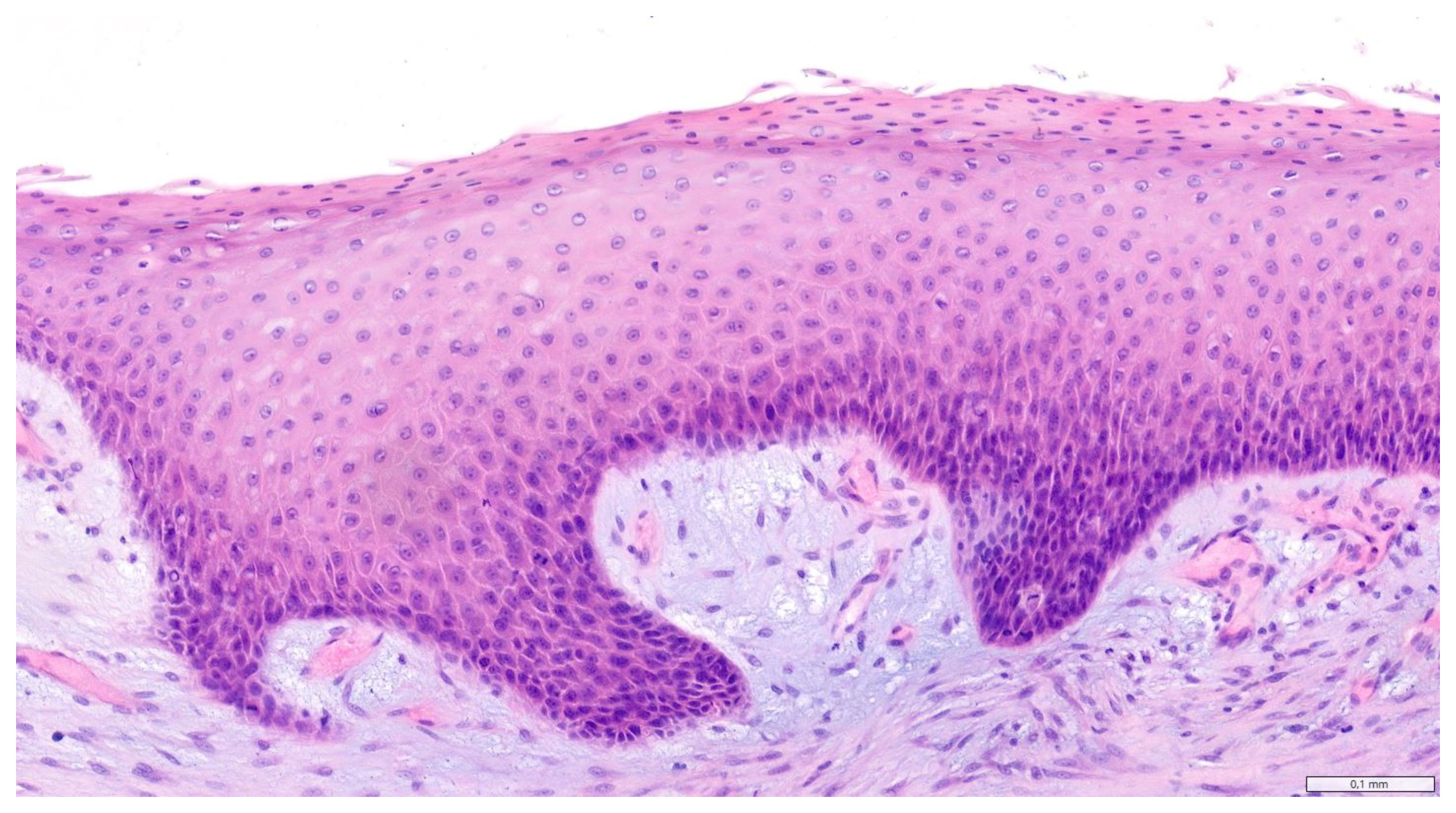

Supplement: Supplementary file 9 — (JPG 3.49 MB) [file 428_2025_4252_MOESM9_ESM.jpg]

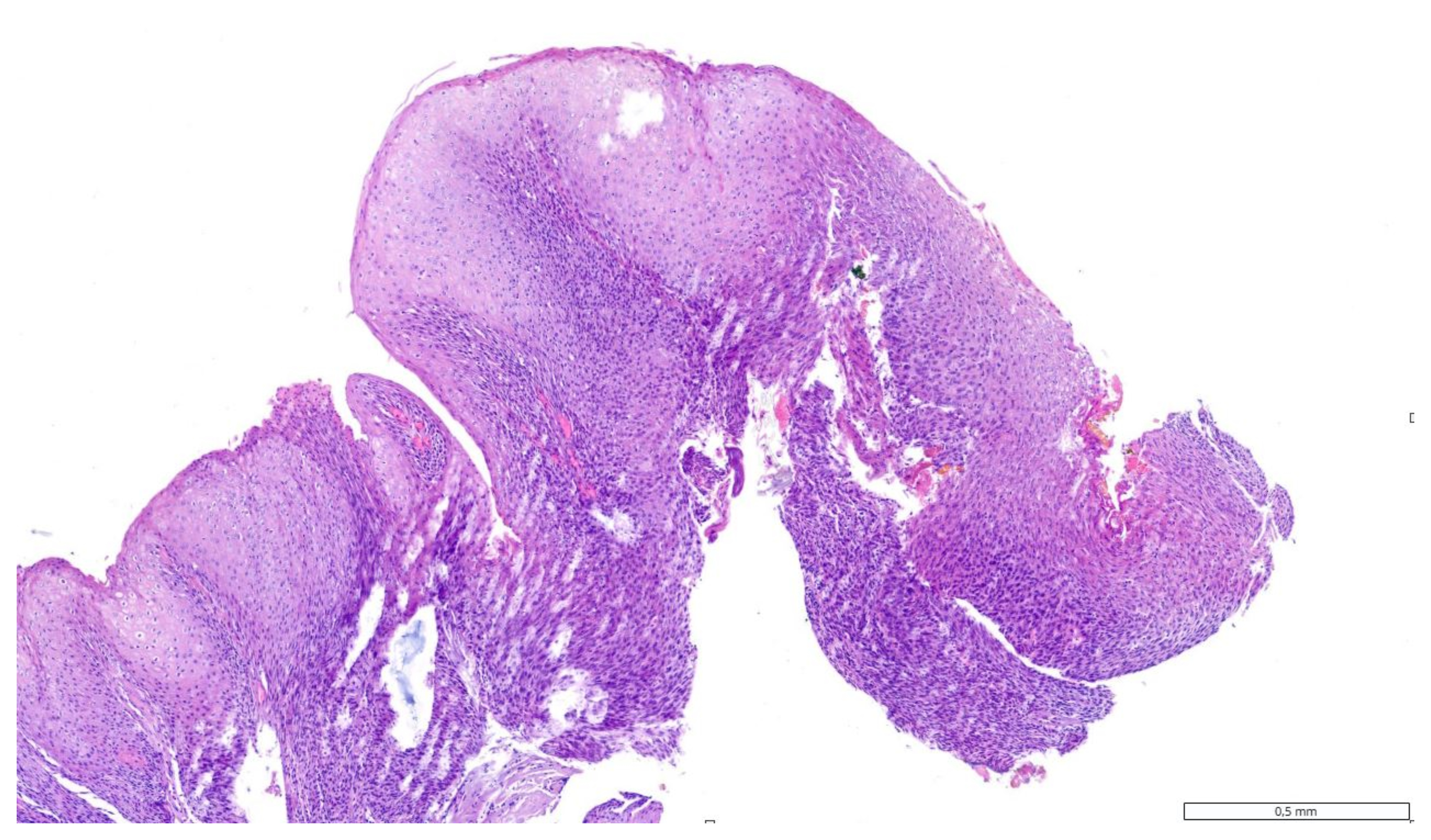

Supplement: Supplementary file 10 — (JPG 3.04 MB) [file 428_2025_4252_MOESM10_ESM.jpg]

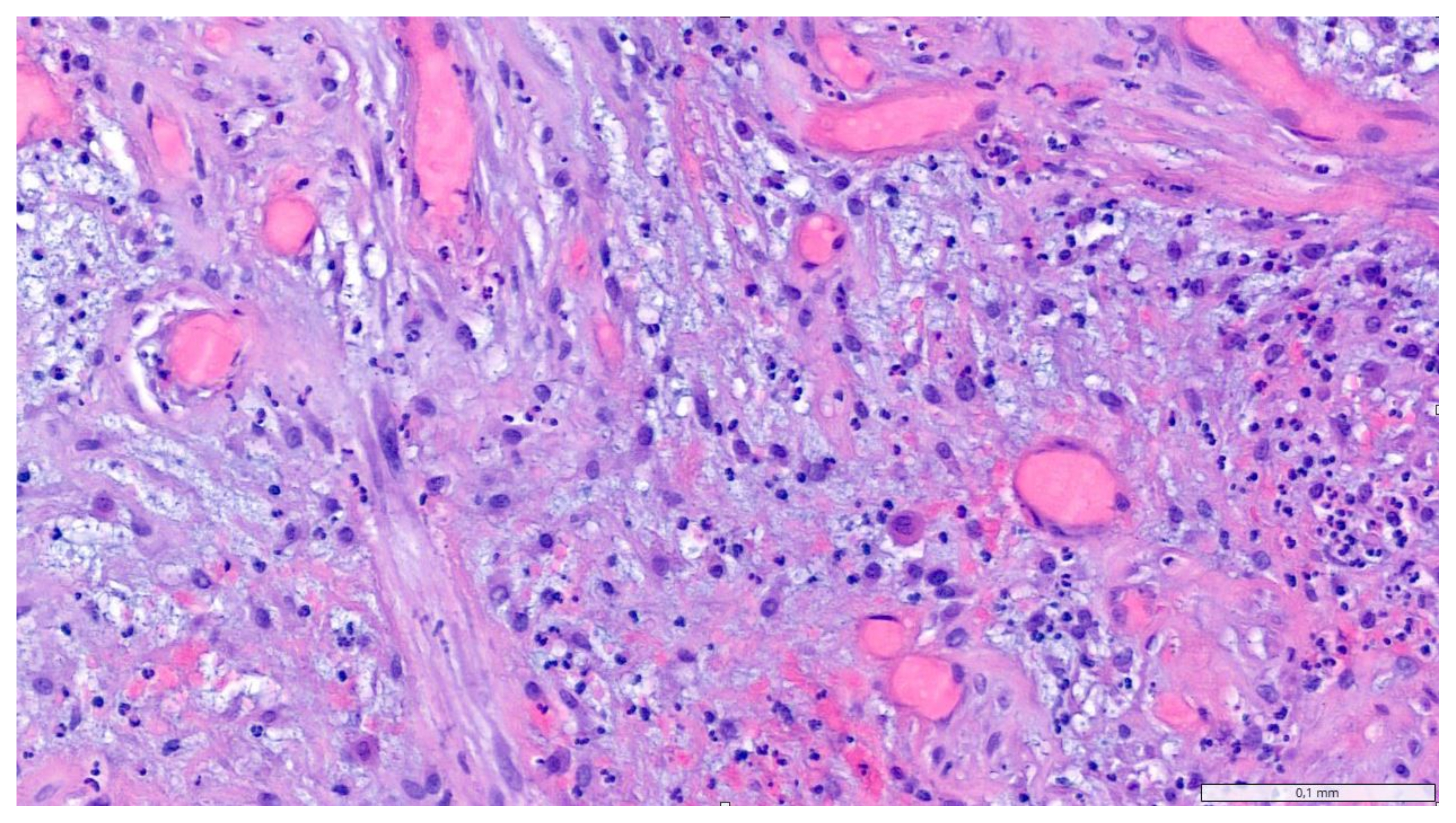

Supplement: Supplementary file 11 — (JPG 4.25 MB) [file 428_2025_4252_MOESM11_ESM.jpg]

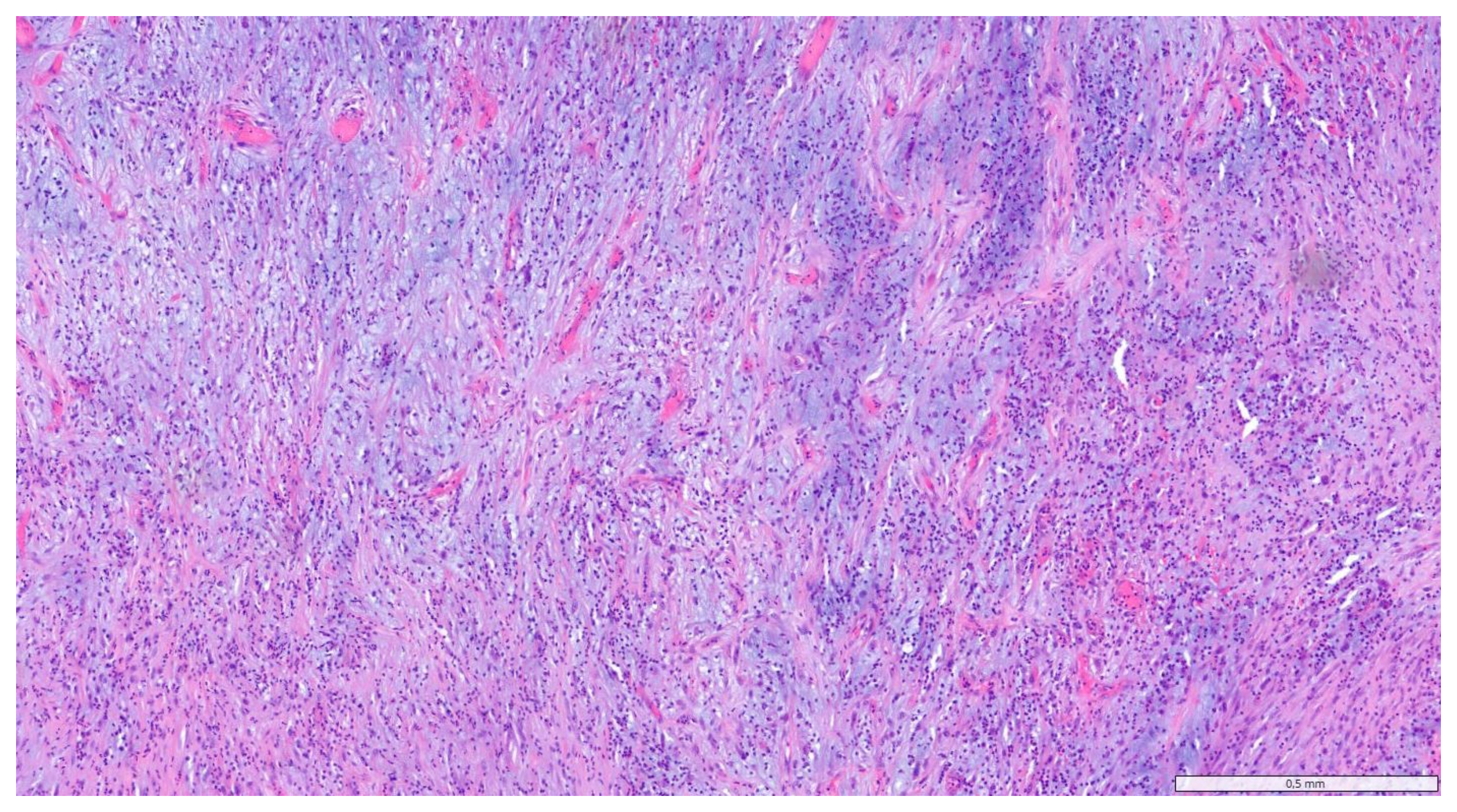

Supplement: Supplementary file 12 — (JPG 5.00 MB) [file 428_2025_4252_MOESM12_ESM.jpg]

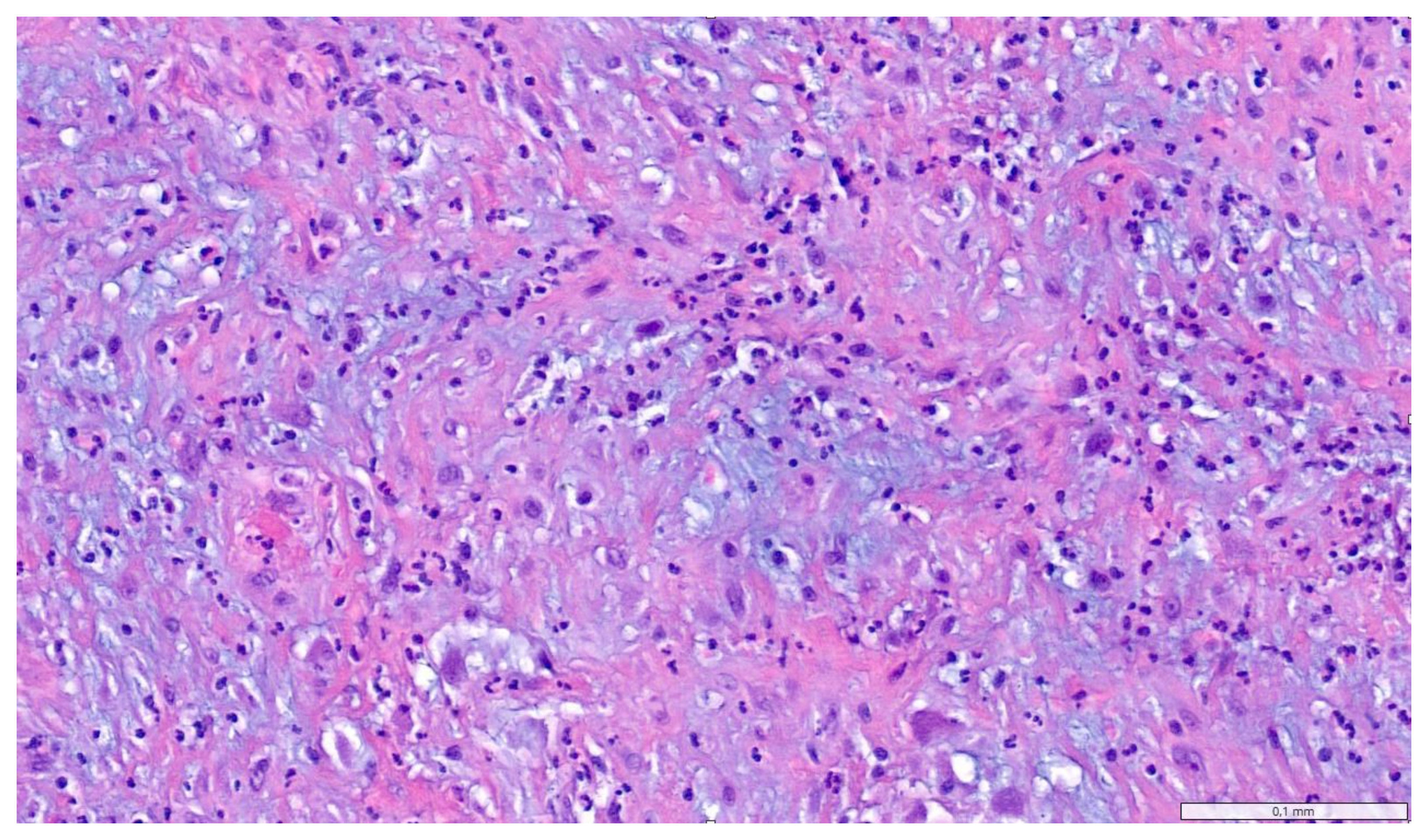

Supplement: Supplementary file 13 — (JPG 4.55 MB) [file 428_2025_4252_MOESM13_ESM.jpg]

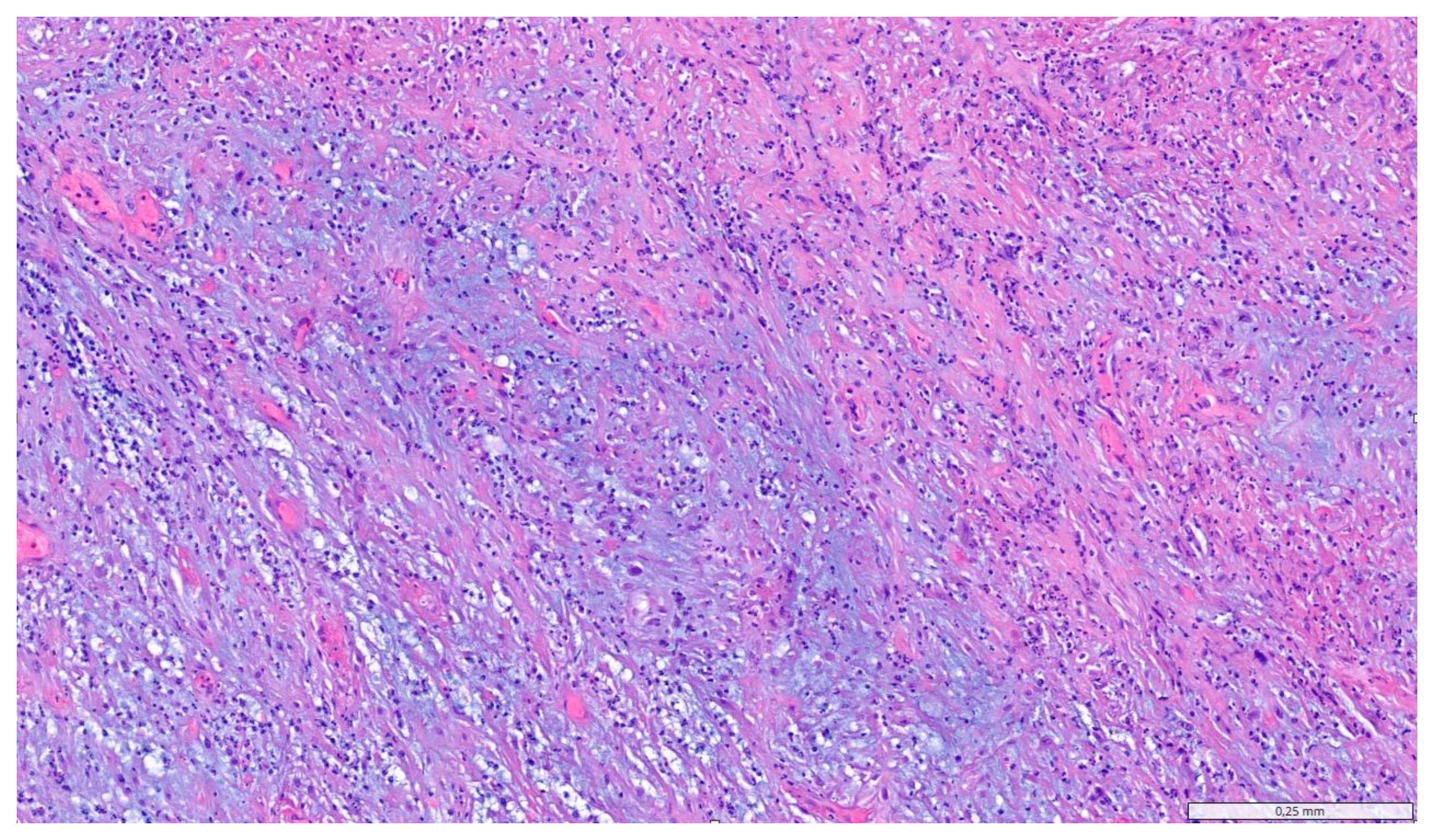

Supplement: Supplementary file 14 — (JPG 5.41 MB) [file 428_2025_4252_MOESM14_ESM.jpg]

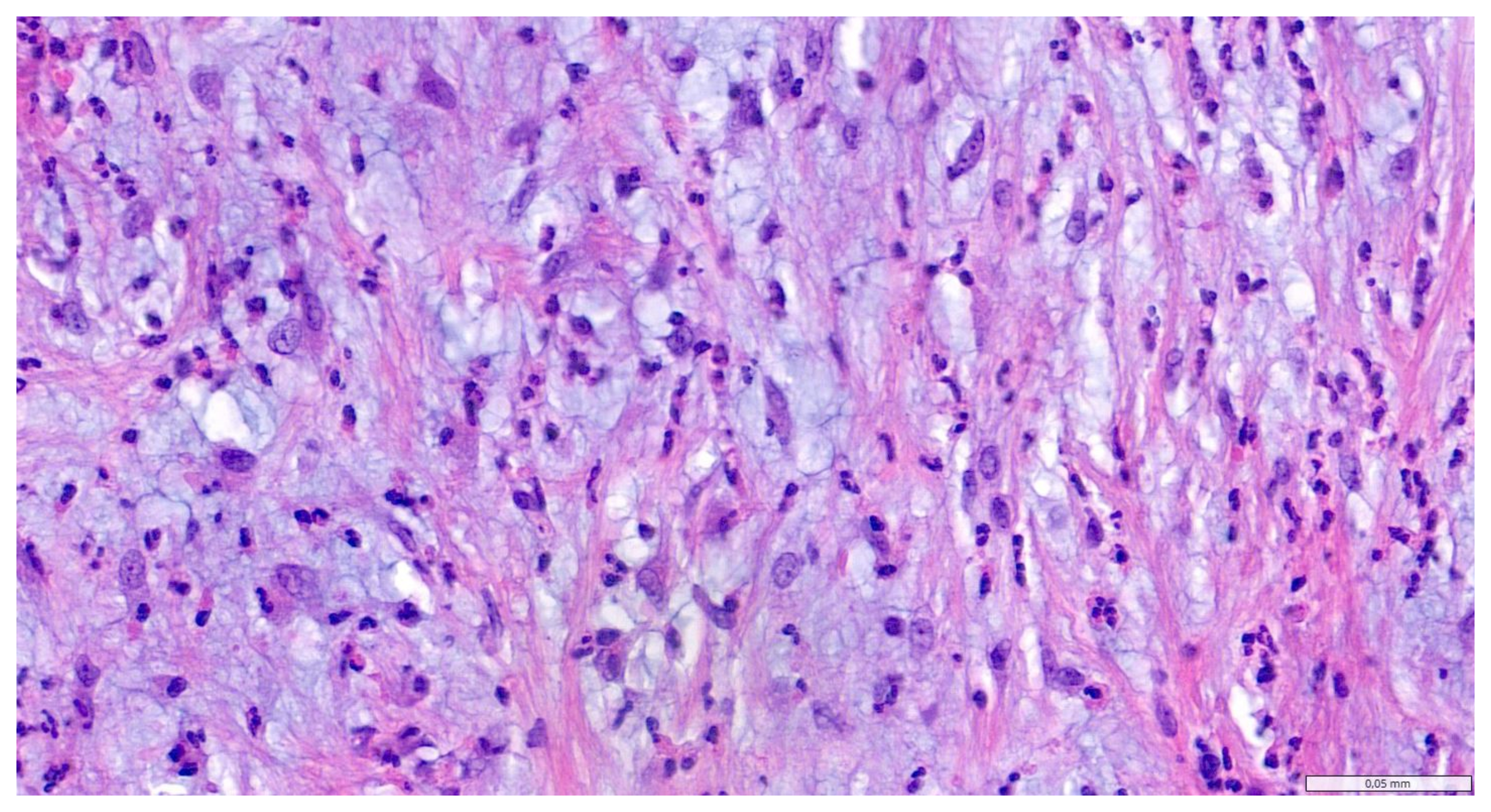

Supplement: Supplementary file 15 — (JPG 4.09 MB) [file 428_2025_4252_MOESM15_ESM.jpg]

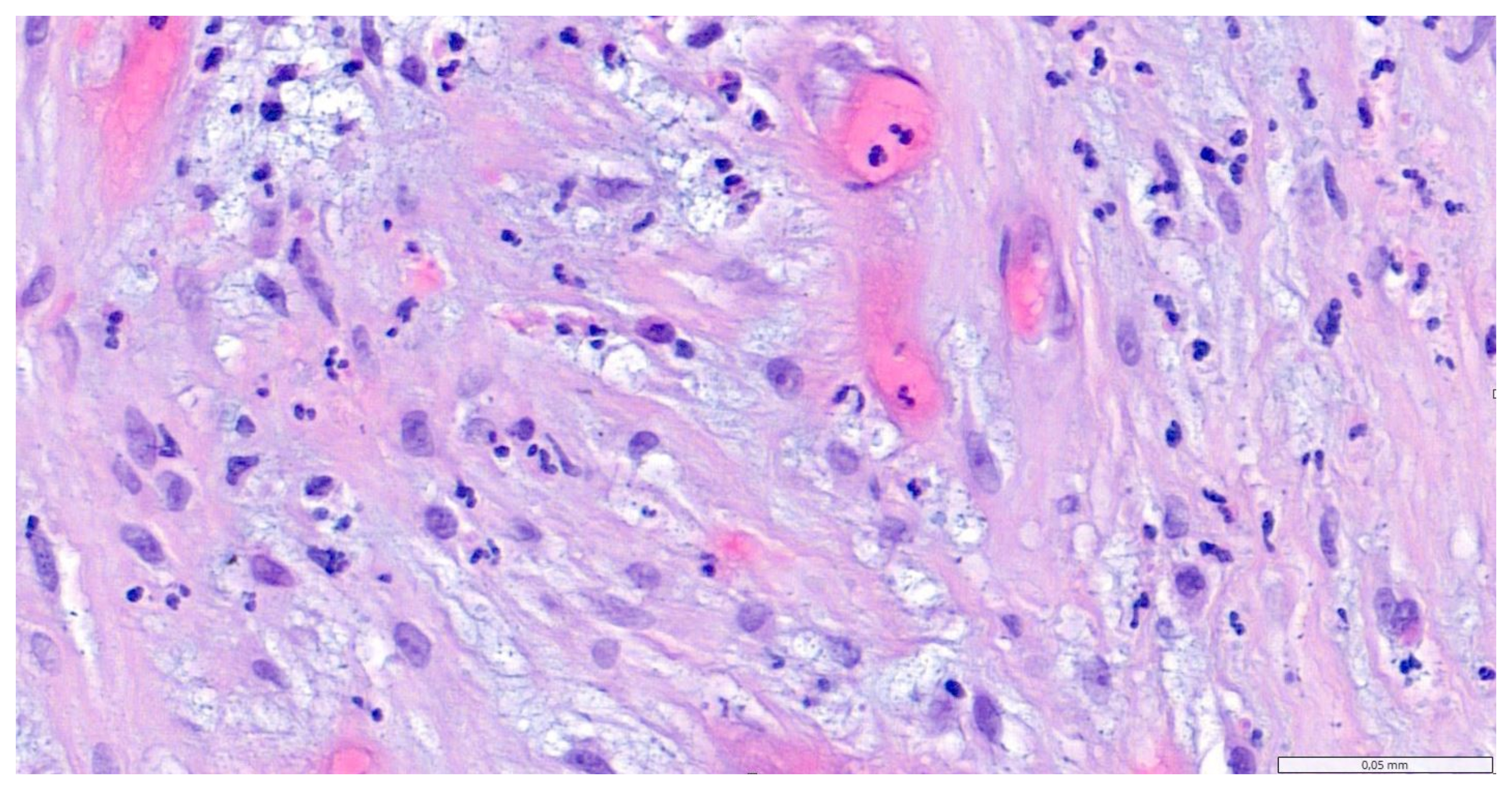

Supplement: Supplementary file 16 — (JPG 3.48 MB) [file 428_2025_4252_MOESM16_ESM.jpg]

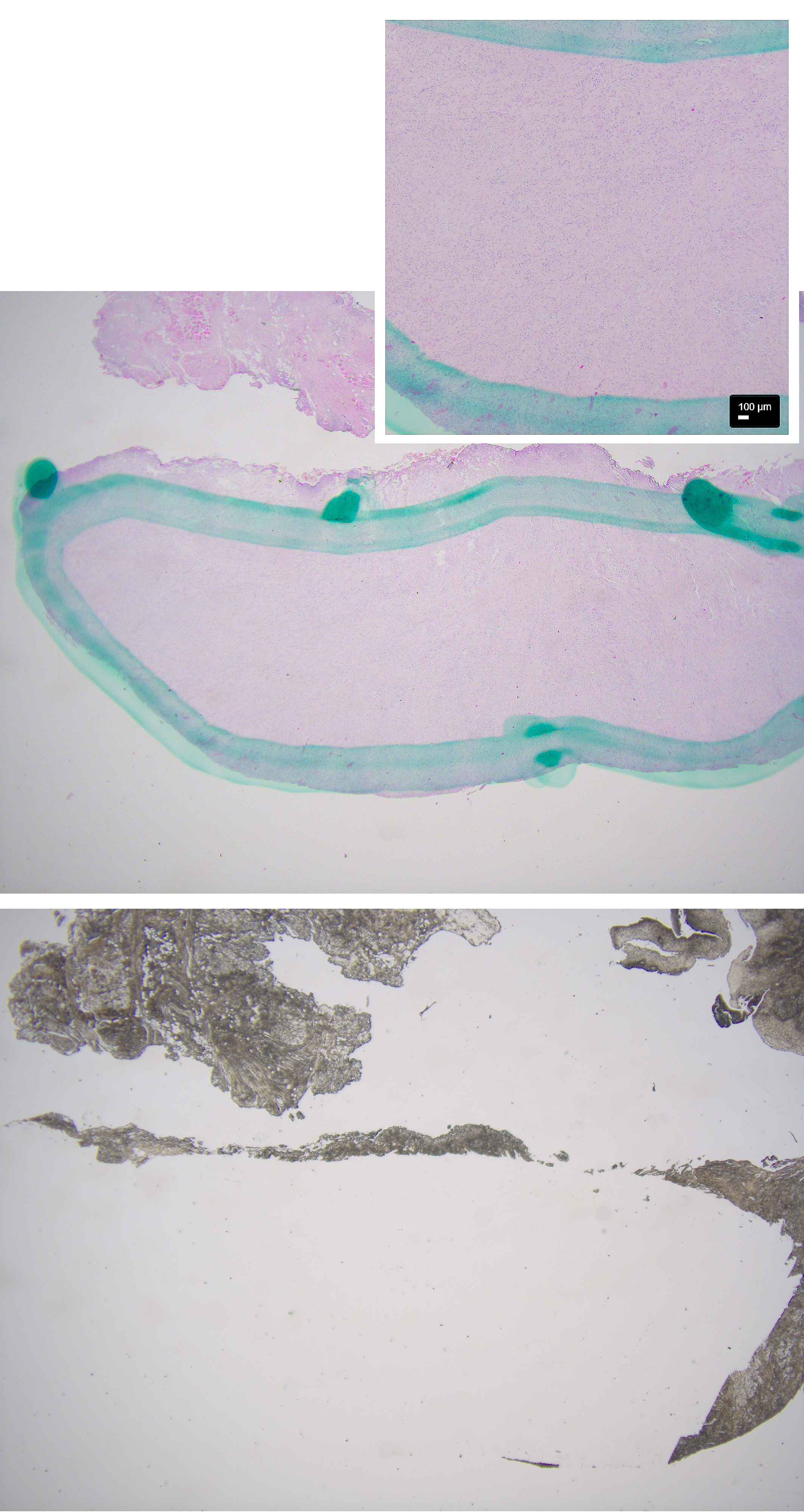

Supplement: Supplementary file 17 — (JPG 7.23 MB) [file 428_2025_4252_MOESM17_ESM.jpg]

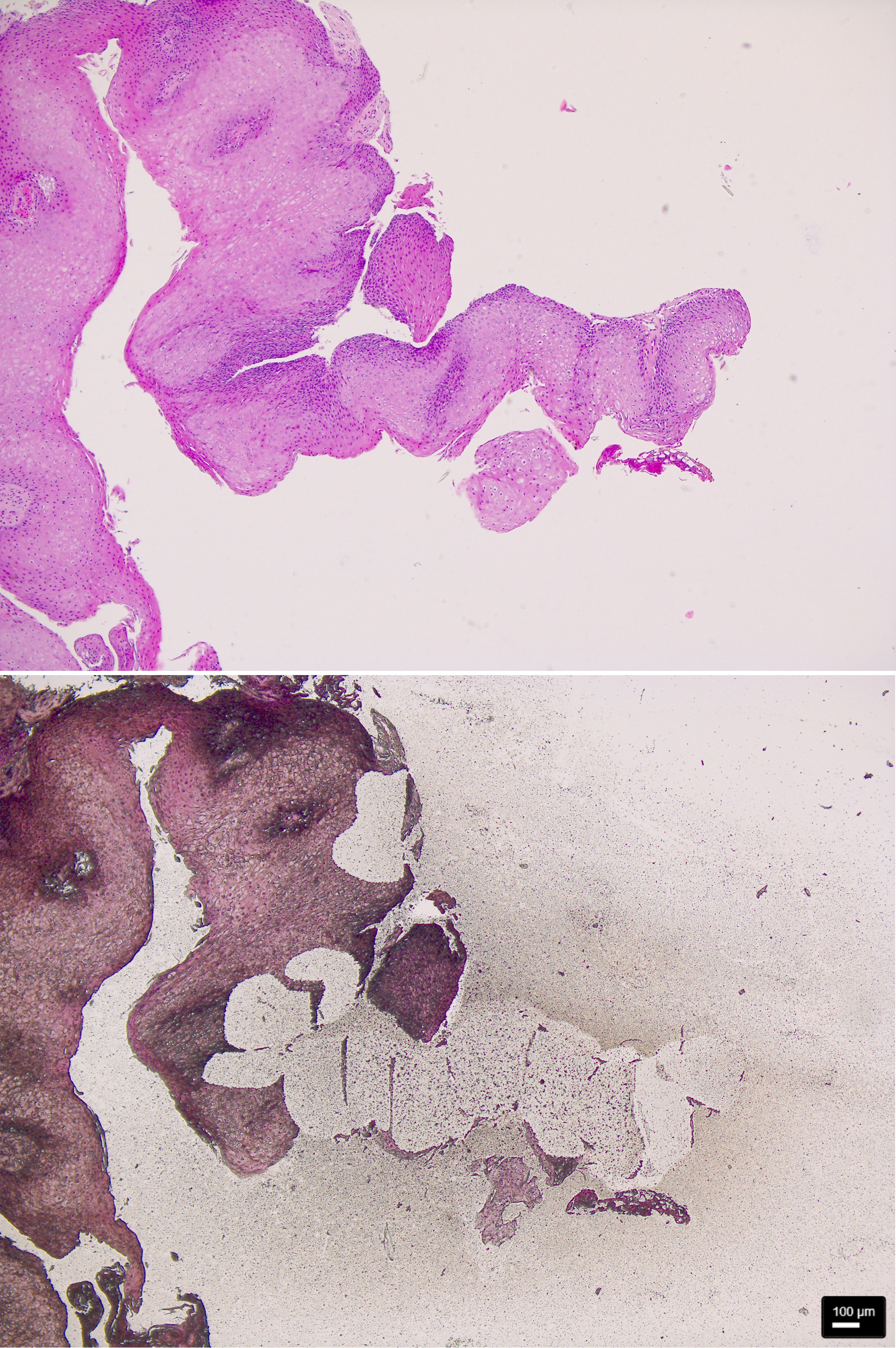

Supplement: Supplementary file 18 — (JPG 6.30 MB) [file 428_2025_4252_MOESM18_ESM.jpg]
